# Mature Microsatellites: Mechanisms Underlying Dinucleotide Microsatellite Mutational Biases in Human Cells

**DOI:** 10.1534/g3.112.005173

**Published:** 2013-03-01

**Authors:** Beverly A. Baptiste, Guruprasad Ananda, Noelle Strubczewski, Andrew Lutzkanin, Su Jen Khoo, Abhinaya Srikanth, Nari Kim, Kateryna D. Makova, Maria M. Krasilnikova, Kristin A. Eckert

**Affiliations:** * Intercollege Graduate Program in Genetics, Huck Institutes of Life Sciences, Pennsylvania State University College of Medicine, Hershey, Pennsylvania 17033; ‡ Department of Pathology, Gittlen Cancer Research Foundation, Pennsylvania State University College of Medicine, Hershey, Pennsylvania 17033; § Department of Biochemistry and Molecular Biology, Pennsylvania State University College of Science, University Park, Pennsylvania 16802; † Graduate Program in Bioinformatics and Genomics, Huck Institutes of Life Sciences, Pennsylvania State University, University Park, Pennsylvania 16802; ** Department of Biology, Pennsylvania State University, University Park, Pennsylvania 16802; †† Center for Medical Genomics, Pennsylvania State University, University Park, Pennsylvania 16802

**Keywords:** short tandem repeats, mismatch repair, microsatellite instability, strand slippage

## Abstract

Dinucleotide microsatellites are dynamic DNA sequences that affect genome stability. Here, we focused on mature microsatellites, defined as pure repeats of lengths above the threshold and unlikely to mutate below it in a single mutational event. We investigated the prevalence and mutational behavior of these sequences by using human genome sequence data, human cells in culture, and purified DNA polymerases. Mature dinucleotides (≥10 units) are present within exonic sequences of >350 genes, resulting in vulnerability to cellular genetic integrity. Mature dinucleotide mutagenesis was examined experimentally using *ex vivo* and *in vitro* approaches. We observe an expansion bias for dinucleotide microsatellites up to 20 units in length in somatic human cells, in agreement with previous computational analyses of germ-line biases. Using purified DNA polymerases and human cell lines deficient for mismatch repair (MMR), we show that the expansion bias is caused by functional MMR and is not due to DNA polymerase error biases. Specifically, we observe that the MutSα and MutLα complexes protect against expansion mutations. Our data support a model wherein different MMR complexes shift the balance of mutations toward deletion or expansion. Finally, we show that replication fork progression is stalled within long dinucleotides, suggesting that mutational mechanisms within long repeats may be distinct from shorter lengths, depending on the biochemistry of fork resolution. Our work combines computational and experimental approaches to explain the complex mutational behavior of dinucleotide microsatellites in humans.

Microsatellites, short tandem repeat DNA sequences with base unit sizes ranging from 1 to 6 base pairs, are abundant in both intragenic (introns, exons, untranslated regions) and intergenic regions of the human genome (Li *et al.* 2002). Approximately 17% of human genes contain microsatellite repeats within open reading frames (Gemayel *et al.* 2010), and intragenic microsatellites can play a prominent role in regulating gene expression and protein function (Li *et al.* 2004; Gemayel *et al.* 2010). In this study, we focus on the mutational behavior of dinucleotide microsatellites. Allele-length polymorphisms at specific dinucleotide microsatellite loci are implicated as genetic risk factors in a number of diseases. For example, the length of a polymorphic [GT/CA] allele within intron one of the EGFR gene is inversely correlated with transcription (Gebhardt *et al.* 1999), and EGFR expression is increased in breast tumors with [GT/CA]_15_ alleles, relative to tumors with [GT/CA]_18_ alleles (Buerger *et al.* 2000, 2004). Length changes of a [GT/CA] allele in the eNOS gene affect splicing regulation and as a result are associated with the risk of coronary artery disease (Stangl *et al.* 2000; Hui *et al.* 2005). Mutation of dinucleotides within exons is expected to directly affect protein sequence and potentially also function; of importance, instability of exonic dinucleotides within 14 cancer-associated genes was detected in tumors of head and neck squamous cell carcinoma patients (Wang *et al.* 2012).

A defining characteristic of microsatellites is their dynamic mutational behavior and high level of germline polymorphism among individuals (Ellegren 2004). Based on changes in mutational behavior, we have defined the threshold length at which a short tandem repeat becomes a microsatellite (Kelkar *et al.* 2010; Ananda *et al.* 2013). The threshold length for dinucleotides defined in this manner is five units (10 bp). The major factors influencing microsatellite mutability are specific to the microsatellite itself; these intrinsic factors include motif size, motif composition, and the overall number of units in the microsatellite (Kelkar *et al.* 2008; Eckert and Hile 2009). Recently, we demonstrated that distinct cellular mechanisms might contribute to dinucleotide microsatellite mutability at different repeat length ranges before and after the threshold length (Ananda *et al.* 2013). In a previous comparative genomics study of microsatellite mutability, we observed distinct phases of mutability as a function of allele length (Kelkar *et al.* 2008). Together, these observations argue for unique mutation mechanisms within microsatellites of lengths above the threshold.

Genome-wide studies have identified directional biases in the mutational behavior of long microsatellites. Early studies of human germline mutations at dinucleotide microsatellites indicated that expansions outnumber contractions (Ellegren 2000). Subsequent studies, based on larger data sets, also demonstrated an expansion bias, with a contraction bias seen only for very long alleles (Huang *et al.* 2002; Sun *et al.* 2012). These latter studies are also consistent with computational modeling interrogating human dinucleotide microsatellites using their genomic distributions (Calabrese and Durrett 2003) or human-chimpanzee interspecific comparisons (Sainudiin *et al.* 2004). A recent study found a pattern of an expansion and contraction biases for tetranucleotide alleles (Sun *et al.* 2012) that is similar to the one observed for dinucleotide microsatellites. Thus, the directional biases that exist for in microsatellites in the human genome seem to depend on their repeat number (length). Computational models have been derived that extend the stepwise mutation model to assume higher mutation rates at long microsatellites (Bell and Jurka 1997), to allow different rates of expansions and deletions depending on length (Whittaker *et al.* 2003), to impose an upper limit on allele sizes (Feldman *et al.* 1997), to incorporate occasional mutations involving a large number of repeated units (Di Rienzo *et al.* 1994), or to integrate slippage and point mutations depending on microsatellite allele length (Kruglyak *et al.* 1998). Clearly, understanding the mutational mechanisms operating within long microsatellite alleles is necessary to correctly model the evolution of these sequences.

Several potential mechanisms may underlie mutational biases within microsatellites, including DNA polymerase errors during synthesis and postreplication mismatch repair (MMR) [reviewed in (Eckert and Hile 2009)]. MMR proteins act in multiple DNA metabolic pathways to modulate mutagenesis: the canonical pathway, which removes base-base mispairs and insertion/deletion loops (IDLs) generated during DNA synthesis; homologous recombination and double-strand break repair pathways; and DNA damage signaling pathways (Lazzaro *et al.* 2009; Pena-Diaz and Jiricny 2012). With the use of mouse models, a complex effect of MMR on both the germline and somatic mutability of very long, disease-associated trinucleotide microsatellites has been demonstrated (McMurray 2010). In these studies, MMR proteins differentially affect expansion and contraction (deletion) mutations in a manner that is dependent on both the identity of the specific MMR protein and the sequence of the microsatellite (van den Broek *et al.* 2002; Dragileva *et al.* 2009; Bourn *et al.* 2012).

Replication fork stalling and/or the perturbation of DNA repair synthesis caused by non-B DNA structures formed within microsatellites also is correlated with expansion of disease-related microsatellites in several model systems [reviewed in (Pearson *et al.* 2005; Mirkin and Mirkin 2007; Wells 2007)]. Replication fork stalling has been demonstrated primarily at expanded trinucleotide microsatellites (Samadashwily *et al.* 1997; Pelletier *et al.* 2003; Krasilnikova and Mirkin 2004b; Voineagu *et al.* 2009). Replication perturbations have been proposed to generate repeat expansions in several models, which include replication fork stalling followed by fork reversal (Sogo *et al.* 2002) and fork stalling followed by template strand switching events (Shishkin *et al.* 2009). The extent to which dinucleotide microsatellite repeats lead to replication fork stalling has not been previously investigated.

The goals of this study were several fold. First, we sought to understand the biological significance of long dinucleotide microsatellites by analyzing their locations within the human genome and identifying the genes harboring such microsatellites within exonic sequences. Second, we quantified the types of mutational biases present in human cells using direct experimental approaches. Third, we tested possible mechanisms influencing the mutational behavior of mature dinucleotide microsatellites by using both *ex vivo* and *in vitro* assays. Fourth, we examined whether replication fork progression is affected by the presence of long dinucleotide microsatellite alleles. We find that mature microsatellites up to 20 units, present in numerous protein-coding regions, display a mutational bias toward expansion that is lost in the absence of mismatch repair. Additionally, long dinucleotide repeats do stall replication fork progression, which may affect mutational mechanisms. Our results have uncovered unexpected mechanistic parallels between dinucleotide microsatellites commonly found within the human genome and rare, expanded trinucleotide microsatellite alleles.

## Materials and Methods

### Gene Ontology (GO) enrichment analysis

We obtained the set of dinucleotide microsatellites from the reference human genome (hg19) by using custom scripts [see (Ananda *et al.* 2013) for details]. Using Galaxy (Giardine *et al.* 2005; Blankenberg *et al.* 2010; Goecks *et al.* 2010), we intersected these microsatellites with a list of exons obtained from the UCSC Genome Browser (Kent *et al.* 2002; Karolchik *et al.* 2008). The list of exonic microsatellites was then combined with HUGO gene annotations (HUGO Gene Nomenclature Committee at the European Bioinformatics Institute) to map each exonic microsatellite to the associated gene. Next, using functions in the R package ‘GOstats’ (Falcon and Gentleman 2007), we investigated whether the mature microsatellite-containing genes were enriched for specific GO functional annotations (in comparison to the genes in the remainder of the genome). Specifically, we used hyperGTest function (with a *P*-value cut-off of 0.01, and ontology specified as “molecular function”), which uses a hypergeometric test to compare the two gene sets (mature microsatellite-containing genes *vs.* all other genes in the genome) and determines an over/underrepresentation of GO “molecular function” annotations in a selected gene set (mature microsatellite-containing genes in our case).

### Reagents

Antibiotics and 5-fluoro-2’-deoxyuridine (FUdR) were purchased from Sigma-Aldrich Co. (St. Louis, MO). Fetal bovine serum was purchased from Hyclone Laboratories, Inc. (Logan, UT) and gentamycin was purchased from Mediatech, Inc. (Manassas, VA). Recombinant DNA polymerase β (pol β) was purified as described (Opresko *et al.* 1998).

### Cell lines

LCL721 cells are an Epstein-Barr virus (EBV)-transformed cell line derived from the B lymphocytes of a clinically normal female donor (Kavathas *et al.* 1980). LCL1261 cells are an EBV-transformed cell line derived from the B lymphocytes of a patient with Turcot syndrome and are PMS2 deficient (Parsons *et al.* 1995). Cells were cultured in buffered RPMI 1640 supplemented with 10% (LCL721) or 15% (LCL1261) fetal bovine serum and 50 mg/mL gentamycin. The MMR protein expression profile of LCL721 and LCL1261 cell lines has been previously reported (Shah and Eckert 2009). HCT116 cells were derived from a human colorectal carcinoma and are deficient in MLH1 and MSH3 (Bennett *et al.* 1997). The HCT116+chr3 cell line has been complemented with an additional chromosome 3 to restore MLH1 gene function (Koi *et al.* 1994). Both HCT116 cell lines were cultured in buffered Dulbecco’s modified Eagle medium + F12 supplemented with 10% fetal bovine serum and 50 mg/mL gentamicin. Culture medium for HCT116+chr3 cells also contained 400 µg/mL Geneticin. The presence or absence of MMR proteins in the HCT116/HCT116+chr3 cell lines was confirmed by immunoblot analyses (data not shown). Replication analysis of plasmids was performed in two mammalian cell lines: 293A (purchased from Invitrogen/Life Technologies, Grand Island, NY) and COS-1 cells (purchased from Sigma-Aldrich, St. Louis, MO). COS-1 cells and 293A cells were grown in Dulbecco’s modified Eagle medium supplemented with 10% newborn calf serum (COS-1) or fetal bovine serum (293A).

### Vector construction

The herpes simplex virus thymidine kinase type 1 (HSV-*tk*) gene-containing vector, pSStu1, is a derivative of the pGem3Zf (-) phagemid and has been previously described (Eckert *et al.* 2002a; Hile and Eckert 2008). The oriP-tk shuttle vector (pJY1) contains the HSV-*tk* gene and the oriP replication origin sequence from EBV (Hile *et al.* 2000). The psGSV-*tk* shuttle vector contains the HSV-*tk* gene and the replication origin sequence from Simian Virus 40 (SV40 ori). psGSV-*tk* was constructed by amplifying the SV40 ori from pEGFP-N1 and cloning the amplified product into the *Xba*I site of pGTK4, followed by *Bam*HI digestion and religation to remove the chloramphenicol resistance marker. HSV-*tk* gene cassettes containing microsatellite alleles were constructed by inserting tandem repeats in-frame between bases 111 and 112 of the target HSV-*tk* gene, in the sequence context GT^TCTC, as described previously (Eckert *et al.* 2002a,b; Kelkar *et al.* 2010) HSV-*tk* gene cassettes containing various microsatellites were subcloned from the pSStu-based vector into pJY1 and psGSV-tk shuttle vectors. Several subclones were isolated and re-analyzed to confirm wild-type HSV-*tk* function and DNA sequence. For microsatellite replication stalling assays, oligonucleotides containing [AT/TA]_n_, [TC/AG]_n_, [GT/CA]_n_, and [GC/CG]_n_ repeats were first cloned and elongated in pYES plasmid, as described (Krasilnikova and Mirkin 2004b). Plasmid pUCneoH was obtained by inactivating the existing *Hin*dIII site of pUCneo, and insertion of oligonucleotides containing *Hin*dIII at the blunt-ended AatII site. Microsatellite-containing pUCneoH plasmids were obtained by inserting the corresponding repeat-containing *Hin*dIII fragments of pYES into the *Hin*dIII site of pUCneoH.

### OriP-*tk* shuttle vector assay

Shuttle vector constructs containing the various microsatellite alleles were introduced into LCL populations by electroporation, and cells containing shuttle vector DNA were selected by the presence of 300 μg/mL (LCL721) or 100 μg/mL hygromycin (LCL1261) for 5−7 d, and maintained with 150 μg/mL and 50 μg/mL, respectively, as described (Eckert *et al.* 2002b; Shah and Eckert 2009). Briefly, selected cell populations were cloned by serial dilution, and individual clones were expanded to a population size of ~2−3 × 10^8^ cells. An alkaline extraction method was used to purify shuttle vector DNA, which was used to electroporate *Escherichia coli* strain FT334. To select for HSV-*tk* mutant plasmids, bacteria were plated in the presence of 50 μg/mL chloramphenicol (Cm) and the absence or presence of 40 μM FUdR. FUdR selects for bacteria harboring a plasmid with any mutation that inactivates the HSV-*tk* gene. The HSV-*tk* mutant frequency is defined as the number of FUdR^R^ + Cm^R^ colonies divided by the total number Cm^R^ colonies. The mutation rate was estimated by dividing the observed HSV-*tk* mutant frequency by number of cell population doublings between the time of cloning and shuttle vector DNA extraction, as described (Eckert *et al.* 2002b), for each human cell clone. To derive specific microsatellite mutation rates, the DNA sequence changes of 20−30 independent FUdR^R^ Cm^R^ mutants were determined from several human clones. The proportion of mutants arising within the microsatellite region (not within the HSV-*tk* gene coding region) was multiplied by the estimated mutation rate for each clone to calculate the specific mutation rate of the microsatellite. Statistical differences in the mutation rates observed among various shuttle vector sequences were analyzed using nonparametric tests and mutation rates derived for at least three human cell clones per vector. Statistical analyses of mutational biases were performed by pooling microsatellite mutations within a given vector observed among all cell clones, followed by Fisher exact test (two-sided).

### SV40-tk shuttle vector assay

For each template, ten 75-cm^2^ plates were transfected with 4 µg each of psGSV-tk and pPVU-o, a plasmid containing SV40 T Antigen. Cells were grown for 3 d before harvest. Cell pellets were washed with phosphate-buffered saline and then frozen at −80°. Plasmids were extracted from the cells using QIAGEN Plasmid Minikit (Valencia, CA). Dpn1 digestion was performed to isolate plasmids that have undergone complete replicated in human cells. For mutational analyses, *Eco*RV and *Mlu*I digestion was performed to isolate the HSV-*tk* mutational target sequence and the resulting fragment was hybridized to gapped DNA heteroduplex molecules, as described (Eckert *et al.* 1997). Successful gap hybridization was verified by gel electrophoresis, and the resulting hybrid molecules were electroporated into *E. coli* FT334 followed by selective plating for mutational analyses, as described previously. The HSV-*tk* mutant frequency was determined after two independent transfections for each cell line. Independent mutants were isolated and sequenced to derive the mutational specificity.

### *In vitro* DNA polymerase assay

Linear DNA fragments and single-stranded DNA (ssDNA) were prepared from pSStu vectors and used to construct gapped duplex molecules for each construct, as described (Eckert *et al.* 2002a; Hile and Eckert 2008). The *in vitro* reactions contained 1 pmol of primed ssDNA template at 40 nM concentration and 10 pmol of pol β. Two independent polymerase reactions were performed for each tandem repeat-containing template, as described (Eckert *et al.* 2002a). The polymerase mutant frequency for each strand of the microsatellite (*e.g.*, GT *vs.* CA) was determined separately from two independent polymerase reactions per strand. To control for pre-existing mutations present within the DNA synthesis template, we determined the HSV-*tk* mutation frequency for each ssDNA by electroporation of FT334, followed by selective plating on media containing 250 µg/mL carbenicillin, with or without FUdR (Eckert *et al.* 2002a). For each template strand, the polymerase error frequency was calculated by subtracting the ssDNA background mutation frequencies from the observed pol β HSV-*tk* mutation frequencies. To determine the polymerase error frequency (Pol EF) within each microsatellite target region, a mutational spectrum of 20−30 mutants was generated for each template, using mutants isolated from two independent polymerase reactions per template. The Pol EF for a specific microsatellite allele was calculated from the proportion of the microsatellite insertion/deletion (indel) mutants (among the total sequenced), multiplied by Pol EF for each template. The Pol EF for each microsatellite allele (*e.g.*, GT/CA_10_) was estimated by adding the Pol EF for each strand (*e.g.*, GT_10_ + CA_10_). Statistical differences in the observed mutational specificities among the microsatellite alleles were analyzed using the χ^2^ or Fisher exact test and the numbers of mutants in each class (summed for both strands).

### Replication pausing assays

Two different assays were performed to analyze the first and subsequent replication cycles of plasmids. For the analysis of the first replication cycle, 293A cells were transfected with plasmids using Lipofectin (Invitrogen) according to the manufacturer’s instructions and lysed after 6 hr. To analyze the replication of plasmids in the subsequent replication rounds, COS-1 cells were transfected using Turbofect (Fermentas, Glen Burnie, MD) according to manufacturer’s protocol. The cells were grown for 30 hr before isolation of replication intermediates. Isolation of replication intermediates from mammalian cells and their analysis via two-dimensional neutral/neutral gel electrophoresis were performed as described (Krasilnikova and Mirkin 2004a; Voineagu *et al.* 2009).

## Results

**Figure 1:**
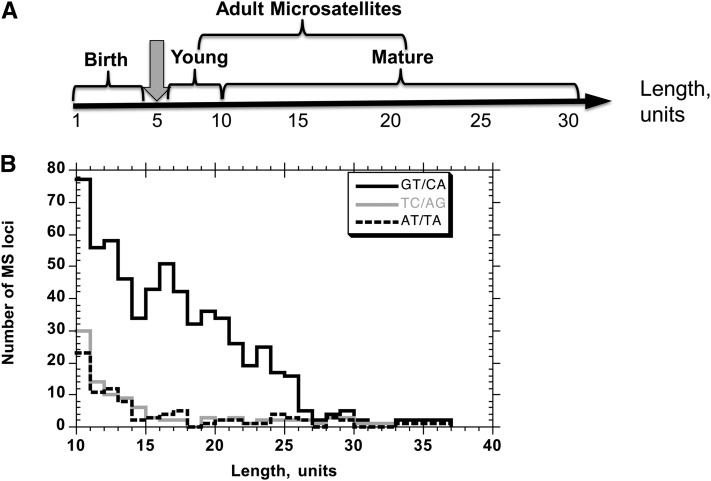
Dinucleotide microsatellites in the human genome. (A) Definition of mature microsatellites. (B) Distribution of genic mature dinucleotide microsatellites in the human genome (hg19) as a function of motif sequence. Solid black line, GT/CA motifs; gray line, TC/AG motifs; dashed line, AT/TA motifs. Only one mature length exonic GC/CG motif (11 units) was identified (not shown).

The focus of this study is mature dinucleotide microsatellites in the human genome, which are expected to be at increased risk for mutation. Here, we define mature microsatellites as pure tandem repeats, longer than the microsatellite threshold, that are not expected to contract to lengths at or below the threshold in a single mutational event. We and others have described the microsatellite lifecycle in three phases: birth, when a locus acquires the necessary numbers of repeats to attain the threshold length; adulthood, a dynamic phase when a locus is above the threshold for mutagenesis; and death, when the locus repeat length dips below the threshold (Amos and Rubinstzein 1996; Buschiazzo and Gemmell 2006; Kelkar *et al.* 2010). Adult microsatellites can be further divided into “young” and “mature” based on the ability of the microsatellite to degrade below the threshold in one replication cycle (Figure 1A). For dinucleotides, the threshold was determined to be five units (Kelkar *et al.* 2010; Ananda *et al.* 2013). We empirically defined mature dinucleotides as repeats 10 units or greater in length, as we and others have not observed mutational events greater than four unit deletions in previous studies of dinucleotide microsatellites (Kelkar *et al.* 2010; Ananda *et al.* 2013). Therefore, dinucleotides of 10 units are not expected to contract to lengths below the threshold.

### Mature dinucleotide microsatellites in the human genome

We computed the number of mature length dinucleotide alleles in the reference human genome (hg19) as a function of a region’s gene annotations. There are 83,840 dinucleotide loci with ≥10 repeats (mature microsatellites), of which 35,654 are present within genes. Of these genic dinucleotide loci, 34,831 (97.7%) are intronic and 823 (2.4%) are exonic. Among mature dinucleotide microsatellites located in exons, the predominant motif is [GT/CA]_n_ (636 alleles), followed by [TC/AG]_n_ and [AT/TA]_n_ (with 95 and 91 loci, respectively). Only one mature-length exonic [GC/CG] locus is present in the human genome (of 11 units). Given that the human polymorphism incidence measured for dinucleotides of 10 units in length was ~40% (Ananda *et al.* 2013), we expect that mature microsatellites will be highly polymorphic in human populations, when not under selective pressure. Unfortunately, as shown in our recent study (Ananda *et al.* 2013), we cannot reliably measure human polymorphism rates at dinucleotides above 10 repeat units (or 20 bp) from resequencing projects, due to limitations imposed by the short read lengths of current datasets.

Dinucleotide loci as long as 37 units (74 bp) in length are present within exons (Figure 1B). Mature dinucleotides are present in 450 exons within the human genome and correspond to 385 genes (as annotated by HUGO; Supporting Information, Table S1). We examined whether these 385 genes are significantly enriched for specific functions categories by comparing GO terms for this set *vs.* these for genes in the remainder of the genome (Table 1). We found that genes with mature length, exonic dinucleotide microsatellites are significantly enriched (*P* ≤ 0.01) for several GO functional terms, including transcription factor activity and sequence-specific DNA binding; ion binding; various channel activities; protein domain specific binding; and GTPase regulator activity (Table 1).

**Table 1:** GO molecular functions significantly overrepresented in dinucleotide microsatellite containing genes

GOMFID*^a^*	Term	Number of Genes in Term*^b^*	Number Expected by Chance	Number With Mature Dinucleotide Repeats	*P*-Value*^c^*
GO:0005488	Binding	8250	151.9	173	0.0003
GO:0043565	Sequence-specific DNA binding	614	12.4	26	0.0003
GO:0033130	Acetylcholine receptor binding	3	0.1	2	0.0012
GO:0000014	Single-stranded DNA specific endodeoxyribonuclease activity	4	0.1	2	0.0024
GO:0022838	Substrate-specific channel activity	391	7.9	17	0.0025
GO:0005097	Rab gtpase activator activity	51	1	5	0.0035
GO:0022803	Passive transmembrane transporter activity	406	8.2	17	0.0037
GO:0003828	Alpha-N-acetylneuraminate alpha-2,8-sialyltransferase activity	5	0.1	2	0.0039
GO:0022836	Gated channel activity	307	6.2	14	0.0039
GO:0030695	Gtpase regulator activity	410	8.3	17	0.0041
GO:0019899	Enzyme binding	518	10.4	20	0.0043
GO:0031404	Chloride ion binding	77	1.6	6	0.0046
GO:0005244	Voltage-gated ion channel activity	189	3.8	10	0.0051
GO:0003700	Transcription factor activity	961	19.4	31	0.0066
GO:0019904	Protein domain specific binding	329	6.6	14	0.0071
GO:0035258	Steroid hormone receptor binding	39	0.8	4	0.0076
GO:0046872	Metal ion binding	4189	84.4	104	0.0079
GO:0016018	Cyclosporin A binding	7	0.1	2	0.008
GO:0030169	Low-density lipoprotein binding	21	0.4	3	0.0082
GO:0043167	Ion binding	4290	86.5	106	0.0084

GO, Gene Ontology.

a Genes associated with each of these GO terms are listed in Table S1.

b Some genes are cross listed in multiple terms. For example, genes included in the specific category “metal ion binding” are also counted in the general category “binding.”

c Only significant enrichments (*P* < 0.01) are listed in this table.

Our computational analyses have defined a set of genes that are potentially at high risk for mutational inactivation due to microsatellite length variation. In the following sections, we analyze mature dinucleotide microsatellite mutagenesis in human cells to elucidate the mechanisms underlying their mutability.

### Mutation rate and directional biases in immortalized human cells

**Figure 2:**
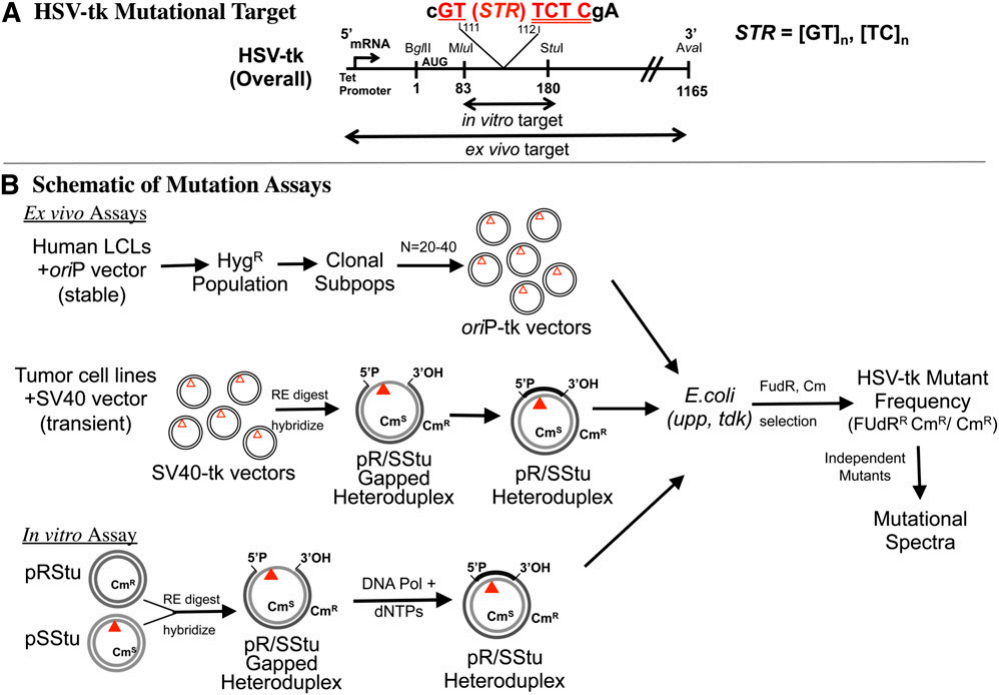
Schematic of the HSV-*tk* experimental system. (A) HSV-*tk* mutational target. Short tandem repeat (STR) sequences were inserted in-frame between bases 111 and 112 of the HSV-*tk* mutational target to create artificial microsatellites. Inactivating mutations can arise within the MS sequence, as well as within either the entire HSV-*tk* promoter and gene sequence (*ex vivo* assay) or an ~100 base-pair region (*Mlu*I−*Stu*I) of the HSV-*tk* gene (*in vitro* assay). (B) Illustrations of the mutagenesis approach. Top line: *Ex vivo* oriP-*tk* shuttle vector assay. MS-containing HSV-*tk* gene cassettes were cloned into the EBV-oriP derived pJY1 shuttle vector (Hile *et al.* 2000). The location of the MS sequences is indicated by an inverted triangle. Human lymphoblastoid cell lines (LCL) were electroporated with shuttle vector, and vector-bearing populations were selected using hygromycin. Clonal subpopulations were isolated by limiting dilution, and expanded ~20−40 cell generations. Episomal DNA was extracted and shuttle vector purified. Middle line: SV40-tk shuttle vector assay. MS-containing HSV-*tk* gene cassettes were cloned into the SV40 ori, psGSV shuttle vector. HCT116 and HCT116+chr3 cells were transfected with shuttle vector and pPVU-o (contains SV40 T-antigen). After 3 d, plasmids were harvested from cells. Fragments containing replicated mutational targets were digested with restriction endonucleases and hybridized to gapped heteroduplex molecules. Bottom line: *In vitro* DNA polymerase assay. Gapped heteroduplex molecules were created by hybridizing the *MluI*-*Stu*I large fragment from the pRStu vector to ssDNA derived from pSStu vectors. Gel-purified gapped substrates were used as templates for DNA synthesis reactions containing purified human DNA polymerases. In all three assays, product DNAs (purified oriP-tk shuttle vectors or gap-filled heteroduplexes) were introduced into *E. coli* (upp, tdk) for mutational analyses. Cm selects for bacteria bearing the shuttle vector, or bacteria derived from the heteroduplex Cm^R^ strand; FUdR selects for HSV-*tk*-deficient bacteria. DNA sequence changes of independent FUdR^R^ mutants are determined to derive a mutational spectrum for each MS vector.

We previously reported the use of HSV-*tk* gene cassettes containing in-frame insertions of defined microsatellite sequences to study the relationships between DNA sequence and microsatellite mutagenesis in mitotic human cells (Figure 2A) (Hile *et al.* 2000; Eckert *et al.* 2002b). Forward mutational analyses allow the quantitation of mutation rates in the artificial microsatellites after vector isolation and genetic selection in *E. coli*. Our established *ex vivo* assay (Figure 2B) uses an *ori*P-based episomal shuttle vector system to study microsatellite mutagenesis during stable DNA replication in EBV-transformed cell lines. Using the *ori*P system, we directly measured mutation rates of mature microsatellites, and tested whether mutational biases exist among dinucleotide microsatellites of varying length and sequence composition. The mutation rate of a series of [GT/CA]_n_ or [TC/AG]_n_ microsatellite alleles ranging in length from 10 to 20 units was measured after stable replication in the non-tumorigenic LCL-721 cell line. (We are unable to analyze mutagenesis at [AT/TA]_n_ motifs of mature length using our experimental system due to high background mutation frequencies, so this motif was not analyzed in this assay.) The individual microsatellite (MS) mutation rates for each clonal population, determined after DNA sequence analyses of independent mutants are presented in Table S2.

**Figure 3:**
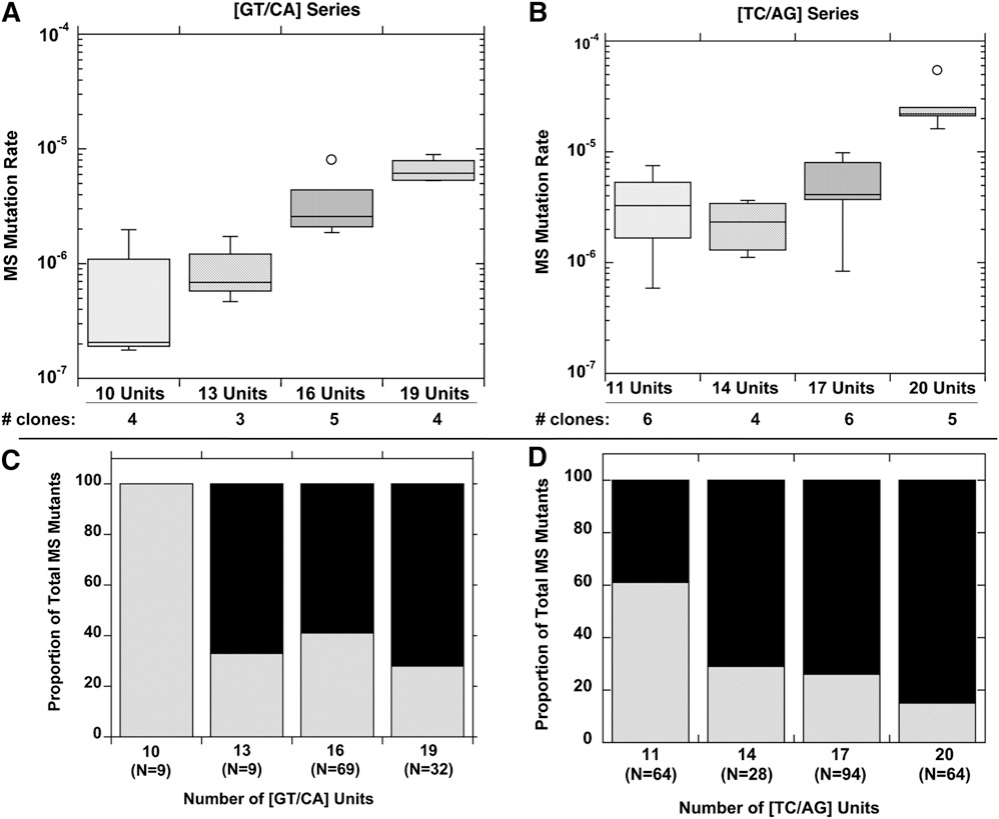
Microsatellite mutation rates and directional biases observed in nontumorigenic, mismatch repair-proficient human lymphoblastoid cells. The oriP-tk shuttle vector assay was performed using LCL-721 cells. (A) MS mutation rate (mutation frequency per cell generation) as a function of GT/CA units. Data are box plots for the number of clones indicated. (B) MS mutation rate as a function of TC/AG units. Data are box plots for the number of clones indicated. (C) Mutational biases within GT/CA alleles as a function of length. Total number of MS mutants observed among all clones is shown in parentheses for each allele. Black bars, expansions of 1 or more units; gray bars, deletion of one or more units (D) Mutational biases within TC/AG alleles as a function of length. Data for the [TC/AG]_17_ allele are taken from reference (Eckert *et al.* 2002b). Black bars, expansions of 1 or more units; gray bars, deletion of one or more units.

As expected from previous studies (Kelkar *et al.* 2010), we observed an increase in MS mutation rate with increasing allele length (Figure 3). For the [GT/CA]_n_ series, as the length of the allele increased ~2-fold, from 10 to 19 units, the median MS mutation rate increased ~30-fold (Figure 3A), a difference that is statistically significant (*P* = 0.0002, Kruskal-Wallis test). In comparison, the MS mutation rate of the [TC/AG]_n_ series increased sevenfold between 11 and 20 units (Figure 3B), a difference that also is statistically significant (*P* = 0.0052, Kruskal-Wallis test). Interestingly, the [TC/AG]_n_ MS mutation rates did not change substantially over a span of five [TC/AG] units: 3.3 × 10^−6^, 2.3 × 10^−6^, and 4.1 × 10^−6^ for lengths of 11, 14, and 17 units respectively. In contrast, the median MS mutation rates of the [GT/CA]_n_ alleles increased progressively, with a 3-fold change from 10 to 13 units (2.1 × 10^−7^ and 6.9 × 10^−7^, respectively) and an additional 5-fold change to 26 × 10^−7^ at 16 units. However, when similar repeat numbers are compared, the [TC/AG]_n_ alleles are usually more mutable than the [GT/CA]_n_ alleles. For example, the rate for a [TC/AG]_11_ allele is 16-fold greater than that for the [GT/CA]_10_ allele (*P* = 0.038, Mann-Whitney test), whereas the rate for a [TC/AG]_20_ allele is ~4-fold greater than that of a [GT/CA]_19_ allele (*P* = 0.016, Mann-Whitney test). Given the slope of the [GT/CA]_n_ *vs.* unit number median mutation rate curve (not shown, derived from data in Figure 3A), the lower mutability of the [GT/CA] alleles than the [TC/AG] alleles cannot be accounted for by the one unit differences in total allele lengths.

Computational studies have suggested that there is a directionality bias in germline dinucleotide microsatellite mutations, such that expansions are more likely than deletions (Amos and Rubinstzein 1996; Ellegren 2000; Amos 2010). We analyzed our data set for the directionality of mutations occurring at a microsatellite allele, namely unit-based expansions *vs.* deletions. In somatic human cells, we observed that the proportion of expansion mutations is greater than deletion mutations for both dinucleotide motifs at all except the shortest alleles tested. For the [GT/CA]_n_ series, no expansion mutations were observed at the shortest length examined (10 units), whereas greater than 50% of the MS mutations were expansions within the longer alleles (13−19 units; Figure 3C). For the [TC/AG]_n_ series, ~40% of the microsatellite mutations were expansions within the shortest allele (11 units; Figure 3D). Moreover, a statistically significant increase in the proportion of expansion mutations was observed as the allele length increased (14−20 units), relative to the [TC/AG]_11_ allele (*P* < 0.0001 to 0.006, Fisher’s exact test). A full 80% of the microsatellite mutations arising within the [TC/AG]_20_ allele were expansions (Figure 3D).

To summarize, we observed two types of mutation biases in mature microsatellite alleles using our somatic cell mutation assay: greater mutation rates for [TC/AG] than [GT/CA] alleles and a bias toward expansion *vs.* deletion errors in alleles 13 to 20 units in length. To examine the mechanisms underlying these biases, we investigated the roles of DNA polymerase slippage errors and MMR.

### DNA polymerase error specificity within dinucleotide microsatellites

**Figure 4:**
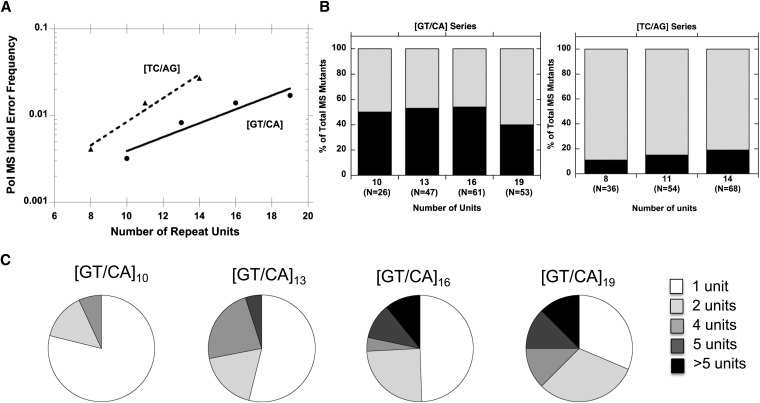
Pol β error rates and specificity within dinucleotide alleles. (A) MS indel error rates as a function of allele length. Error rates were estimated from two independent reactions per template strand. Error rates for each complementary strand were summed to derive the error rate for each allele length. Solid line, GT/CA alleles; dashed line, TC/AG alleles. Lines are an exponential fit of the data. (B) Mutational biases observed for GT/CA and TC/AG alleles, as a function of length. Black bars, expansions of 1 or more units; gray bars, deletion of one or more units. (C) Size distributions of MS indel deletion errors, as a function of allele length.

We used our published *in vitro* DNA polymerase mutagenesis assay (Eckert *et al.* 2002a) to investigate the sources of the sequence composition and directionality biases observed above. Previously, we reported that DNA pol β error frequency for insertion/deletion (Indel) errors within complementary [GT] and [CA] repeats increased ~30-fold from 4 units to 13 units (Kelkar *et al.* 2010). Here, we extended our studies to include [GT/CA]_16_ and [GT/CA]_19_ templates, as well as [TC/AG] templates of 8 to 14 units in length. (Longer [TC/AG] alleles could not be analyzed using the *in vitro* assay due to the high background mutation frequency of the single-stranded DNA preparations.) The relationship between allele length and pol β error rate for unit-based (two nucleotide) Indels is exponential between 10 units and 19 units in length for the [GT/CA] allele (R^2^ = 0.97) and between 8 units and 14 units in length for the [TC/AG] allele (R^2^ = 0.99; Figure 4A). For the alleles tested, the pol β error rates within the [TC/AG] alleles are higher than those within the [GT/CA] alleles of similar length (Figure 4A), similar to the *ex vivo* assay results.

The majority of pol β errors created in both the [GT/CA] and [TC/AG] microsatellite alleles are deletions. Thus, we observed no bias *in vitro* toward the production of expansion mutations in longer [GT/CA] or [TC/AG] alleles (Figure 4B), in contrast to the *ex vivo* assay. Interestingly, we observe a statistically significant increase in the size of pol β deletion errors as the [GT/CA] alleles increase in length, such that 22–25% of deletions were 5 units or more for the [GT/CA]_16_ and [GT/CA]_19_ alleles (Figure 4C; *P* = 0.027, χ^2^ test, 3 d.o.f.). This type of mutation was not observed or rarely observed among microsatellite errors within the [GT/CA]_10_ and [GT/CA]_13_ mutational spectra.

We previously reported that the human DNA polymerase δ also creates a high frequency of Indel errors within the [GT]_19_ microsatellite (Baptiste and Eckert 2012). The mutational specificity bias toward multi-unit [GT] deletions that we report here for pol β is similar to our previous measurements for polymerases δ and κ (Figure S1). Moreover, the specificity of pol β errors within the [TC]_11_ allele (~85% deletions, 15% expansions) is nearly identical to that previously reported for the replicative polymerases, pol α-primase (Hile and Eckert 2004) and pol δ (Hile *et al.* 2012).

In summary, our *in vitro* results show that differential DNA polymerase error rates can contribute to the observed bias regarding motif sequence. However, the specificity of errors created by replicative (pol α, pol δ), repair (pol β), or specialized (pol κ) DNA polymerases cannot readily explain the directionality bias toward expansion mutations with increasing allele length that was observed in human cells.

### Role of MMR in generating a mutational bias toward expansion of [GT/CA]_n_ alleles

In the canonical pathway associated with correction of DNA synthesis errors, MMR proteins recognize premutational intermediates in which the newly synthesized DNA strand (nascent DNA) differs in sequence from the parental DNA strand (template DNA). Failure of MMR to repair the intermediates results in mutations after the next round of DNA synthesis. Microsatellite expansion mutations result from premutational intermediates containing IDLs in the nascent DNA strand, whereas microsatellite deletions result from premutational intermediates containing IDLs in the template DNA strand. Human cells have specification in MMR, which is achieved through the combination of different heterodimers of the MutS and MutL components of the MMR machinery (Pena-Diaz and Jiricny 2012). Human cell lines deficient in one or more MMR proteins have been identified, and we used three such cell lines to examine the role of MMR in generating mature dinucleotide microsatellite mutational bias (Table 2).

**Table 2:** Comparison of [GT/CA]_19_ mutagenesis results from *ex vivo* and *in vitro* assays

	Ex vivo assay				
MMR Heterodimer	LCL721	LCL1261	HCT116+chr3	HCT116	*In vitro* Assay
MutS α	+	+	+	+*^a^*	−
β	+	+	−	−	−
MutL α	+	−	+	−	−
γ	+	+	+	−	−
Expansion MF	4.4 × 10^−6^	7.2 × 10^−3^	8.0 × 10^−4^	4.8 × 10^−3^	5.6 × 10^−3^
Deletion MF	1.7 × 10^−6^	<1.3 × 10^−4^	4.9 × 10^−3^	7.3 × 10^−3^	1.2 × 10^−2^
Expansion:Deletion	2.6:1	>55:1	1:6	1:1.5	1:2

MMR, mismatch repair; MF, #FUdRr + Cmr colonies/Cmr colonies. Expansion MF and Deletion MF were calculated as [MF X (number of expansion or deletion mutants/total number of mutants)].

a Components of the MutSα mismatch recognition complex are present in HCT116 cells but cannot function in MMR because the absence of MLH1 renders the cells devoid of any MutL complex.

First, we examined mutational specificity in the HCT116 human colon cancer cell line, which carries loss-of-function mutations in both the *MLH1* and *MSH3* genes and has been previously shown to be deficient in repair of two-nucleotide loops (Umar *et al.* 1994). Because these cells do not express the MLH1 protein, they are deficient in both MutLα and MutLγ repair complexes. Therefore, although HCT116 cells express MSH2 and MSH6 proteins (MutSα), they are functionally MMR-deficient because they lack a functional MutL heterodimeric complex. We expected that the mutational events observed in HCT116 cells would reflect errors generated by DNA polymerases, which we have shown here and previously to be biased toward deletions. Second, we analyzed mutational biases in HCT116 cells complemented with chromosome 3 (HCT116+chr3). This complementation restores MLH1 protein expression, but does not affect expression of the MSH3 protein. HCT116+chr3 cells are MutSα proficient, but MutSβ deficient. This gain-of-function comparison of HCT116+chr3 cells to the HCT116 parental cell line allows us to determine the role that MSH2/MutSα plays in generating mutational bias. Third, we assessed the role of MutLα in mutational bias using the human lymphoblastoid cell line (LCL1261), which does not express the PMS2 protein, a key component of the MutLα heterodimer. This cell line does express proteins of both the MutSα and MutSβ heterodimers, as well as MLH1 (Shah and Eckert 2009) and MLH3 (data not shown), proteins of the MutLγ complex. A loss-of-function comparison with LCL721 cells allows us to determine the role that PMS2/MutLα plays in generating mutational bias.

To measure microsatellite mutagenesis experimentally in epithelial cells such as HCT116, we modified the *ex vivo* mutational assay by incorporating the SV40 origin of replication (Figure 2B). Three days after transient transfection of the [GT/CA]_19_ construct, plasmids were recovered from HCT116 cells and analyzed for mutation frequency and mutational specificity. As predicted for cells containing no functional MMR complexes, we measured mutant frequencies that are of the same magnitude as those measured in the *in vitro* DNA polymerase assay (Table 2). Of importance, the frequency of expansion mutations generated after replication in MSH3, MLH1-deficient HCT116 cells (4.8 × 10^−3^) is the same as the *in vitro* frequency of pol β expansion errors in the same allele (5.6 × 10^−3^). In addition, no expansion bias is observed after [GT/CA]_19_ replication in HCT116 cells, and the ratio of expansion: deletion mutations is similar to that observed for purified polymerase *in vitro* (Table 2).

**Figure 5:**
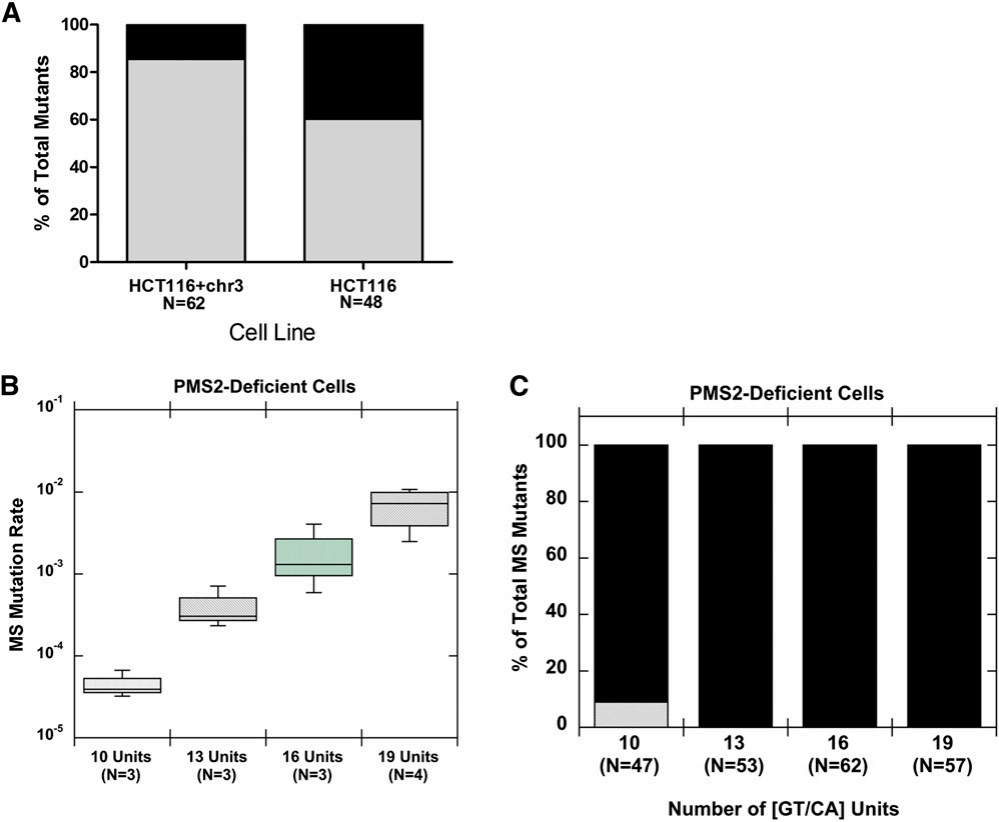
GT/CA microsatellite mutation rates and directional biases observed in mismatch repair-deficient human cells. (A) Mutational biases of GT/CA_19_ microsatellite replication in HCT116+chr3 and HCT116 cells. Black bars, expansions of 1 or more units; gray bars, deletion of one or more units. (B) Mutation rate as a function of allele length determined after oriP-tk shuttle vector replication in PMS2-deficient human lymphoblastoid cells. Data are box plots for the number of clones shown in parentheses. (C) Mutational biases within GT/CA alleles as a function of length. Total number of MS mutants observed among all clones is shown in parentheses for each allele. Solid bars, expansions of 1 or more units; stippled bars, deletion of one or more units.

The mutation frequency of plasmids after replication in HCT116+chr3 cells (5.7 × 10^−3^) was ~2-fold lower than the frequency after replication in HCT116 cells (1.2 × 10^−2^), suggesting that MSH2/MutSα contributes to MMR of replication errors of mature [GT/CA]_n_ dinucleotide repeats. The proportion of microsatellite expansions also was lower for HCT116+chr3 cells (15%) than for HCT116 (40%) cells (Figure 5A). Specifically, we observed that the frequency of expansion mutations was 6-fold lower in HCT116+chr3 cells (8.0 × 10^−4^) than in HCT116 cells (4.8 × 10^−3^) (Table 2). Thus, the presence of MSH2/MutSα suppresses expansion mutations.

We previously demonstrated that MMR mediated by PMS2 (MutLα) also is biased toward repair of expansion mutations within tetranucleotide microsatellites (Shah and Eckert 2009). Here, we tested whether a similar result would be observed within dinucleotide microsatellites. Using the assay *ori*P*-tk* shuttle vector assay, [GT/CA]_n_–containing shuttle vectors were stably replicated in LCL1261 cells. As expected, the observed MS mutation rates were 200- to 1000-fold greater than those measured for MMR-proficient cells (Figure 5B; Table S3). Across all allele lengths examined, we measured a statistically significant 180-fold increase in the median MS mutation rates for the [GT/CA] motif (*P* < 0.0001, Kruskal-Wallis test). Analysis of mutational spectra revealed a striking expansion bias in LCL1261 cells, wherein 91–100% of the microsatellite mutations observed within each [GT/CA] length were expansions (Figure 5C). These results demonstrate that the presence of PMS2 suppresses expansion mutations within dinucleotide alleles. The absolute frequency of expansion mutations in PMS2-deficient LCL1261 cells is similar to that measured in MSH3,MLH1-deficient HCT116 cells (Table 2). Similar to loss of MutSα (comparison of HCT116 + chr3 with HCT116), loss of MutLα (comparison of LCL721 to LCL1261) resulted in an increased frequency and proportion of expansions. Taken together, our data suggest that functional MMR generates directional biases within dinucleotide microsatellites and identifies the MutSα and MutLα complexes as protecting against expansion mutations.

### DNA replication pausing within dinucleotide microsatellites

**Figure 6:**
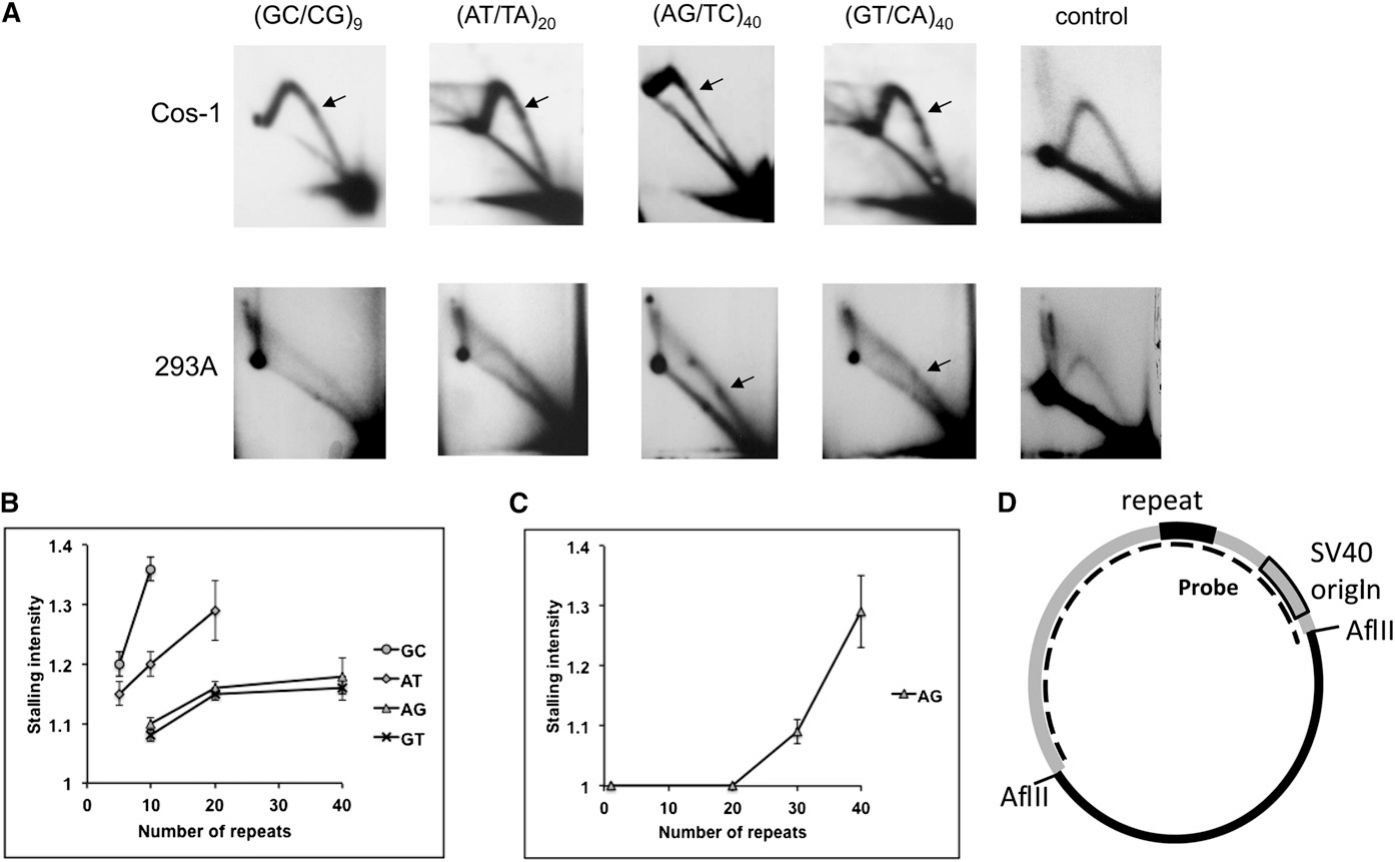
Replication fork stalling within dinucleotide alleles as a function of allele length. (A) Representative two-dimensional gels after replication in Cos-1 (top panels) or HEK293A (bottom panels) cells. (B) Quantitation of pausing in Cos-1 cells. (C) Quantitation of pausing in 293A cells. (D) Schematic of SV40-origin vector indicating position of the microsatellite sequences.

In our next analysis, we considered whether mutational processes in addition to polymerase strand slippage errors and MMR potentially act during DNA replication to produce expansion mutations. Replication fork stalling and template switching mechanisms are well known to correlate with the production of very large trinucleotide repeat expansions (Wells *et al.* 2005; Mirkin and Mirkin 2007). We used an established experimental system (Chandok *et al.* 2012) to analyze whether DNA sequence composition and length affects replication fork progression through mature dinucleotide alleles. To quantitate DNA replication inhibition, [GC/CG]_n_, [AT/TA]_n_, [GT/CA]_n_, and [TC/AG]_n_ alleles of varying lengths were cloned into vectors containing a defined SV40 origin of replication, and DNA replication intermediates isolated from primate Cos-1 cells were analyzed by 2D gel electrophoresis. We observed replication fork stalling at all types of dinucleotide microsatellites, detected as bulges on replication arcs that were not present for the control plasmid (Figure 6A). The position of stalling corresponded to the position of the microsatellite within the plasmid, based on the distance traveled in the first direction of electrophoresis. The strength of the stalling was quantitated by measuring the amount of radioactivity in the bulge, relative to the intensity of the arc (Pelletier *et al.* 2003). Stalling intensity was clearly motif sequence and length dependent (Figure 6B), and a dependence of the stall on motif orientation relative to the replication origin was not observed for any of the repeats (data not shown). At an equivalent number of units, the rank-order for pausing was: [GC/CG] > [AT/TA] > [GT/CA] = [TC/AG] (Figure 6B). We also analyzed dinucleotide microsatellite replication in 293A cells, prior to assembly of a regular chromatin structure. Because 293A cells lack expression of SV40 large T antigen, the plasmid is replicated transiently using an alternative replication mode that initiates randomly throughout the plasmid (Chandok *et al.* 2011). In 293A cells, the only dinucleotide repeat that caused a significant stalling was [TC/AG]_n_ (Figure 6C), where stalling is detectable at a length of about 20 repeated units, and increased with the subsequent increase in the repeat length. Surprisingly, the repeats that have high tendency to form hairpin structures such as [AT/TA]_20_ and [GC/CG]_9_ did not cause stalling in 293 cells (data not shown), although these sequences have a profound effect on SV40-origin dependent replication (Figure 6B).

## Discussion

Microsatellites of 1−6 basepairs per unit are abundant in the human genome, and ~90% of known human genes have microsatellites within exons (Madsen *et al.* 2008). Exonic microsatellites shorter than 33 bp in length were shown to be overrepresented in disease-related genes, particularly cancer and immune system disorders (Madsen *et al.* 2008). Here, we demonstrate that mature dinucleotide microsatellites (20−74 basepairs in length) are present within exons of 385 genes (Table S1) and are significantly enriched in specific classes of genes encoding important biologic activities, such as transcription factor and membrane channel activities (Table 1). Furthermore, we measured a significantly elevated rate of mutation within mature dinucleotide alleles in normal human cells (Figure 3). Therefore, our analysis not only identifies genes potentially at high risk for mutational inactivation due to microsatellite length variation in somatic cells, but also demonstrates the potential functional significance of mature microsatellites and hence the need for a thorough investigation of their mutational behavior.

Our study used various experimental approaches to examine the mutational mechanisms operating within mature microsatellites, which we define as alleles that are not expected to mutate to lengths below or at the threshold for microsatellite mutational behavior in one round of replication. We explored the sources of sequence and directional biases in microsatellite mutability, and our results uncovered several novel facets of dinucleotide mutagenesis. First, our data from nontumorigenic human cells indicate that the [GT/CA]_n_ alleles are usually less mutable than the [TC/AG]_n_ alleles of comparable length (Figure 3), and identify DNA polymerase error rates as the underlying basis of this sequence bias (Figure 4). Second, we demonstrate a directionality bias in somatic human cells that favors expansion mutations for mature dinucleotide microsatellites of 13−20 units (Figure 3). This bias cannot readily be explained by DNA polymerase error biases (Figure 4). Genome-wide studies have identified germline expansion biases in the mutational behavior of dinucleotide microsatellites (Ellegren 2000), with a contraction bias seen only for very long alleles (Huang *et al.* 2002; Sun *et al.* 2012). Possibly, our *ex vivo* data capture the portion of the dinucleotide mutation curve right before an expansion bias switches to a contraction bias. Comparisons of the exact nucleotide range of the bias switch observed in our study in somatic cell mutations and computational studies of human germline mutations (Huang *et al.* 2002; Sun *et al.* 2012) are challenging, because the latter studies report standardized and not actual allele lengths. However, some computational studies indicate this switch occurs at ~20 dinucleotide repeats, although the length at which the switch occurs may be sequence dependent (Calabrese and Durrett 2003; Sainudiin *et al.* 2004).

Third, we found that MMR is a likely source of directional bias within common microsatellites in the human genome. Strikingly, total absence of cellular MMR (Figure 5A) mimics the expansion *vs.* deletion balance seen in our *in vitro* results (Table 2). Comparisons of MutSα-deficient/proficient and MutLα-deficient/proficient cell line pairs identified these two repair complexes as protective against expansion mutations (Table 2). Fourth, we demonstrate that DNA replication fork stalling occurs within mature dinucleotides and is motif and length dependent (Figure 6). Thus, mutational mechanisms within very long dinucleotides may be different than those within shorter alleles (closer to the threshold), depending on the biochemistry of fork resolution/restart.

A constant balance of expansions and contractions of microsatellites may play a role in maintaining their genetic stability over time. Our results show that for dinucleotide alleles in the 13 to 20 repeat number range, expansions occurred more frequently than deletions (Figure 3). The only exceptions seen were the two shortest alleles, [GT/CA]_10_ and [TC/AG]_11_, in the MMR-proficient cells. A bias favoring expansions over deletions within [GT/CA] alleles was previously demonstrated using a yeast model system (Strand *et al.* 1993; Johnson *et al.* 1995). In contrast, we find no directional bias for dinucleotide microsatellite mutations in functionally MMR-deficient HCT116 cells, using a forward assay (Table 2). In fact, the mutational specificity that we observed after replication of the [GT/CA]_19_ vector in HCT116 cells is highly similar to our *in vitro* observations of errors produced by pol β and pol δ at the same microsatellite allele. Our HCT116 cell results using a forward mutation assay differ from the previous conclusions of Yamada *et al.* (2002). One explanation for the different conclusions of the two studies may be the fact that we compared our HCT116 results with chromosome 3-complemented HCT116 cells as a control for MMR, whereas Yamada *et al.* (2002) used MMR-proficient mouse cells as the control comparison. Our complemented cells still lack MutSβ, whereas the mouse cells are presumably proficient for both MutS complexes. Also, mouse cells may replicate and repair looped intermediates differently than human cells.

Our PMS2-deficient cells lack MutLα (MLH1•PMS2) but retain MutLγ (MLH1•MLH3) expression (Shah and Eckert 2009), and likely, activity. Strikingly, of the ~200 independent mutants analyzed from LCL1261 cells deficient in MutLα, only 2% displayed deletion events within the [GT/CA] microsatellite. Our estimated deletion frequency for dinucleotides in LCL1261 cells is ≤ 10^−4^, which is lower than the deletion frequency observed in the fully MMR-deficient HCT116 cell line (Table 2). Therefore, our data indirectly support a role for the MutLγ MMR complex in suppressing deletion mutations within dinucleotide microsatellites. Mouse studies have shown that although *Pms2*^−/−^ (van Oers *et al.* 2010) and *Mlh3*^−/−^ (Chen *et al.* 2005) single knockout mutants both display microsatellite instability and tumor susceptibility, only the double knockout (*Pms2*^−/−^ *Mlh3*^−/−^) mice are indistinguishable from *Mlh1*^−/−^ mice for tumor susceptibility, reduced life span, microsatellite instability, and DNA-damage response (Chen *et al.* 2005). Thus, the two MutL homologs are partially redundant, possibly explaining the 40:1 ratio of *MLH1* to *PMS2* gene mutations seen in Lynch syndrome tumors (Lynch and de la Chapelle 2003; Lynch *et al.* 2009). This redundancy also may help to explain the low prevalence and penetrance of *PMS2* mutations in MMR-deficient colon cancers (Lynch and de la Chapelle 2003) and the reported lack of biochemical data to support MLH3 in Lynch syndrome (Ou *et al.* 2009).

Large expansions of trinucleotide microsatellites are associated with numerous neuromuscular and neurodegenerative disorders, and models involving both DNA repair and replication have been proposed to explain these expansions (McMurray 2010). Although our expansion bias within dinucleotide microsatellites of common lengths in somatic human cells differs qualitatively from massive trinucleotide repeat expansions (we observe only a small number of units added per mutational event), unexpectedly our results uncovered mechanistic parallels to disease-associated trinucleotide expansions. Specifically, we demonstrate that (1) the dinucleotide expansion bias is likely caused by MMR functions, and (2) long dinucleotides cause replication fork stalling in a length- and sequence-dependent manner. We have shown here and previously (Shah and Eckert 2009) that loss of PMS2 in human cells results in a significant bias toward di- and tetranucleotide microsatellite expansion mutations. Recent studies using Friedreich’s ataxia mouse models have demonstrated that PMS2-deficient mice display an increase in expansion mutations within very long [GAA/TCC] microsatellites (Bourn *et al.* 2012; Ezzatizadeh *et al.* 2012). Therefore, PMS2 generally limits expansion mutations within di-, tri-, and tetranucleotide microsatellite alleles. Because pre-mutational IDLs for expansions occur on the nascent strand during DNA synthesis, we propose that PMS2/MutLα-mediated MMR plays a vital role in repairing polymerase errors formed on the nascent strand (Shah and Eckert 2009). This model is not restricted to DNA replication because excision repair, strand break repair, and recombination pathways all require DNA re-synthesis and involve MMR proteins. Indeed, the protective effect of PMS2 toward [GAA/CTT] expansions was suggested recently to occur during the homologous recombination/strand synthesis steps of double strand break repair (Bourn *et al.* 2012). Yeast-null mutants of *RAD27* (*RTH1*) Flap endonuclease, an enzyme required for lagging strand DNA synthesis and DNA repair, display an increased microsatellite mutation frequency and a mutational bias favoring expansion mutations within [GT/CA] microsatellites. These effects are epistatic with MMR gene defects (Johnson *et al.* 1995). MutSβ (Msh2p and Msh3p) has been implicated in promoting trinucleotide repeat expansions in yeast (Kantartzis *et al.* 2012). MutSβ binds to nicks in the lagging strand before Okazaki fragment processing is complete, allowing the repeated sequence to loop out and bind to downstream homologous sequence. When the flap is processed, the loop remains, resulting in an expansion of one to two repeat units. The mutability of very long [GAA/CTT] alleles also is increased in MSH3-deficient mice, with an increase specifically in deletion (contraction) mutations (Ezzatizadeh *et al.* 2012). However, in Huntington’s disease and myotonic dystrophy mouse models, loss of MSH3 decreases somatic mutability of very long [CAG/GTC] and [CTG/GAC] alleles, but has no significant effect on germline mutability or directionality biases (van den Broek *et al.* 2002; Dragileva *et al.* 2009). Future studies are required to examine the specific role of MSH3 in mutational biases of dinucleotide repeats of varying sequence in human cells.

Replication fork stalling and template switching mechanisms correlate with the production of trinucleotide repeat expansions, although a direct cause and effect relationship has yet to be established (Wells *et al.* 2005; Mirkin and Mirkin 2007). We observed replication fork stalling at all dinucleotide microsatellite sequences in a length-dependent manner, suggesting that replication fork restart/template switching mechanisms may also be operative within these regions of the human genome. Our previously developed system (Chandok *et al.* 2012) allowed us to analyze DNA replication fork progression through dinucleotide repeats in two situations: the first replication cycle when the DNA is not completely covered by nucleosomes, and the subsequent replication cycles that occur after a regular chromatin has been established. The various dinucleotide repeat sequences had very different effects on pausing in the first and subsequent replication cycles. Replication fork stalling in the subsequent cycles directly correlated with the hairpin-forming potential of the repeat sequences. In contrast, replication fork stalling in the first cycle was significant only for the [TC/AG] repeat, and at lengths greater than 20 units. Interestingly, we observed a similar replication stalling in the first replication cycle of DNA containing [GAA/CTT] repeats (Chandok *et al.* 2012). Both the [TC/AG] and [GAA/CTT] repeats have the propensity to form H-DNA (triplex) structures. The dependence of fork stalling on repeat length may be explained by the instability of H-DNA structures formed at or within shorter repeats, or the unfavorable energetics of H-DNA formation at shorter repeat lengths, due to a higher proportion of unpaired nucleotides (at triplex borders and in the free strand)(Frank-Kamenetskii and Mirkin 1995).

In summary, our current study has uncovered several new facets regarding the mechanisms underlying dinucleotide microsatellite stability, sequences that are highly abundant in the human genome. First, we demonstrate that the motif sequence-dependent differences in dinucleotide microsatellite mutation rates can be explained by differences in DNA polymerase error rates. Second, a directionality bias favoring expansion of human genome dinucleotide microsatellites has been inferred in computational studies, and our study demonstrates this bias experimentally in human cells, while providing a mechanism to explain the bias. Based on our studies of replicative, repair and specialized polymerases, it is unlikely that the observed directionality bias favoring microsatellite expansion in cells reflects the inherent error specificity of DNA polymerases. We observed that loss of MutSα or MutLα functions promotes directionality biases toward expansion mutations, while total loss of MMR eliminates the expansion bias entirely. Our data raise the possibility that in MMR-proficient cells, IDLs on the template strand are very efficiently repaired by either the MutSβ or MutLγ complex, resulting in a net bias toward expansion mutations, a scenario that could be tested in future studies. Finally, we demonstrate that replication forks are stalled within dinucleotide microsatellites, implicating fork restart and recombination pathways in the stability of long dinucleotide microsatellites. Interestingly, our mechanistic studies of common dinucleotides uncovered mechanistic parallels with the rare trinucleotides, suggesting a complex involvement of MMR in microsatellite genome stability.

## Supplementary Material

Supporting Information
