# Supplementary material for: Mature Microsatellites: Mechanisms Underlying Dinucleotide Microsatellite Mutational Biases in Human Cells
Source: G3 (Bethesda). 2013 Mar 1;3(3):451–63. doi: 10.1534/g3.112.005173 (PMC3583453; doi:10.1534/g3.112.005173)
Supplement: Supporting Information [file supp_3_3_451__index.html]

Supporting Information 

# Mature Microsatellites: Mechanisms Underlying Dinucleotide Microsatellite Mutational Biases in Human Cells

## Supporting Information for Baptiste *et al.*, 2013

**Files in this Data Supplement:**

- Supporting Information - Figure S1 and Tables S1-S3 (PDF, 218 KB)
- Figure S1 - Pol δ (A) and pol κ (B) replicate [GT]19 microsatellite allele with substantial deletion bias (PDF, 166 KB)
- Table S2 - Microsatellite mutation rate and sequence data for independent clones of dinucleotide motifs in LCL721 Cells (PDF, 78 KB)
- Table S3 - Microsatellite mutation rate and sequence data for independent clones in PMS2-Deficient LCL1261 Cells (PDF, 71 KB)
- Table S1 - Genomic coordinates, motifs, length, and gene description for exonic mature dinucleotide microsatellites (.xls, 88 KB)
