# Supplementary material for: Mature Microsatellites: Mechanisms Underlying Dinucleotide Microsatellite Mutational Biases in Human Cells
Source: G3 (Bethesda). 2013 Mar 1;3(3):451–63. doi: 10.1534/g3.112.005173 (PMC3583453; doi:10.1534/g3.112.005173)
Supplement: Supporting Information [file supp_3.3.451_005173SI.pdf]

G3·Genes | Genomes | Genetics

Mature Microsatellites: Mechanisms Underlying Dinucleotide Microsatellite Mutational Biases in Human Cells

Beverly Baptiste[*], Guruprasad Ananda[§], Noelle Strubczewski[**], Andrew Lutzkanin[**], Su Jen Khoo[§§], Abhinaya Srikanth[§§], Nari Kim[§§], Kateryna D. Makova[*** §§§], Maria M. Krasilnikova[§§ §§§], and Kristin A. Eckert[** §§§]

[*] Intercollege Graduate Program in Genetics, Huck Institutes of Life Sciences, Pennsylvania State University College of Medicine, 500 University Drive, Hershey PA 17033
[§] Graduate Program in Bioinformatics and Genomics, Huck Institutes of Life Sciences, Pennsylvania State University, University Park, PA 16802
[**] Department of Pathology, Gittlen Cancer Research Foundation, Pennsylvania State University College of Medicine, 500 University Drive, Hershey, PA 17033
[§§] Department of Biochemistry and Molecular Biology, Pennsylvania State University College of Science, University Park, PA 16802
[***] Department of Biology, Pennsylvania State University, University Park, PA 16802
[§§§] Center for Medical Genomics, Pennsylvania State University, University Park, PA 16802

Corresponding author: Kristin Eckert, 500 University Drive, Mail code H059, Hershey, PA 17033, (717) 531-4065, kae4@psu.edu.

DOI: 10.1534/g3.112.005173

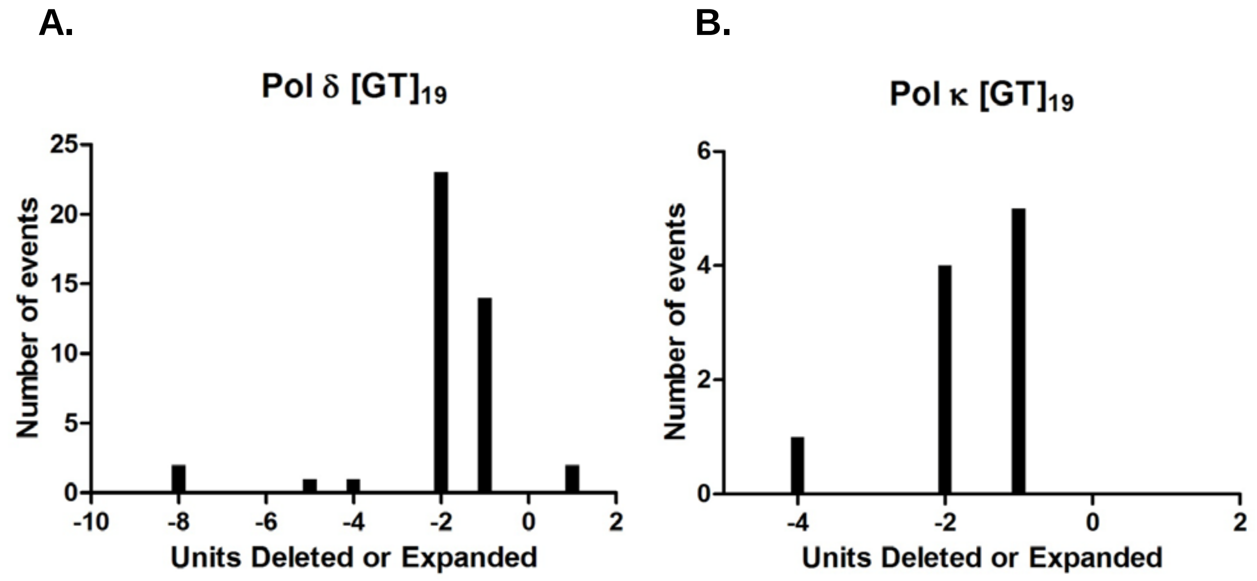

Figure S1 Pol δ (A) and pol κ (B) replicate $[GT]_{19}$ microsatellite allele with substantial deletion bias

Table S1 Genomic coordinates, motifs, length, and gene description for exonic mature dinucleotide microsatellites

Table S1 is available for download at http://www.g3journal.org/lookup/suppl/doi:10.1534/g3.112.005173/-/DC1.

Table S2 Microsatellite mutation rate and sequence data for independent clones of dinucleotide motifs in LCL721 Cells.

[GT/CA] Alleles	GT/CA_{10}				GT/CA_{13}				GT/CA_{16}				GT/CA_{19}			
	Mutation Rate	N[a]	Insertion	Deletion	Mutation Rate	N	Insertion	Deletion	Mutation Rate	N	Insertion	Deletion	Mutation Rate	N	Insertion	Deletion
Clone A	1.97E-06	21	0	6 (5,1)[c]	6.86E-07	21	2 (2,0)	1 (1,0)	4.38E-06	32	6 (6,0)	8 (4,4)	8.92E-06	19	14 (13,1)[d]	0
Clone B	2.08E-07	21	0	1 (1,0)	1.73E-06	22	3 (3,0)	2 (2,0)	8.00E-06	27	9 (9,0)	9 (4,5)	6.92E-06	15	2 (2,0)	4 (4,0)
Clone C	1.77E-07	19	0	1 (1,0)	4.67E-07	21	1 (1,0)	0	2.10E-06	35	12 (12,0)	4 (1,3)	5.21E-06	14	1 (1,0)	2 (1,1)
Clone D	2.05E-07	22	0	1 (1,0)					1.87E-06	9	3 (3,0)	3 (1,2)	5.40E-06	16	6 (6,0)	3 (1,1)[e]
Clone E									2.58E-06	36	11 (11,0)	4 (1,3)				
Totals, by type			83				64				139				64	
Microsatellite			9 (.11)[b]				9 (.14)				69 (.50)				32 (.50)	
HSV-tk coding			11 (.13)				15 (.23)				18 (.13)				10 (.16)	
Rearranged			63 (.76)				40 (.62)				52 (.37)				22 (.34)	

[TC/AG] Alleles	TC/AG_{11}				TC/AG_{14}				TC/AG_{17}				TC/AG_{20}			
	Mutation Rate	N	Insertion	Deletion	Mutation Rate	N	Insertion	Deletion	Mutation Rate	N	Insertion	Deletion	Mutation Rate	N	Insertion	Deletion
Clone A	1.67E-06	12	3 (2,1)	5 (4,1)	3.17E-06	21	6 (6,0)	3 (3,0)	8.01E-06	25	21 (7,14)	1 (1,0)	2.11E-05	19	15 (14,1)	1 (1,0)
Clone B	5.91E-07	22	6 (6.0)	4 (3,1)	3.65E-06	22	3 (3,0)	0	9.80E-06	20	11 (10,1)	3 (3,0)	1.62E-05	20	2 (2,0)	2 (1,1)
Clone C	3.54E-06	14	4 (4,0)	7 (6,1)	1.12E-06	20	5 (5,0)	2 (2,0)	3.71E-06	20	8 (5,3)	6 (6,0)	2.51E-05	14	11 (10,1)	2 (1,0)*
Clone D	2.98E-06	24	5 (4,1)	8 (8,0)	1.49E-06	23	6 (6,0)	3 (3,0)	8.36E-07	14	8 (8,0)	1 (0,1)	5.40E-05	20	12 (11,1)	7 (6,1)
Clone E	7.52E-06	21	6 (5,1)	14 (13,1)					4.09E-06	22	12 (12,0)	8 (8,0)	2.21E-05	30	24 (21,3)	5 (4,1)
Clone F	5.33E-06	30	1 (0,1)	1 (1,0)					4.13E-06	20	10 (10,0)	5 (3,2)				
Totals, by type			123				86				121				103	
Microsatellite			64 (.52)				28 (.33)				94 (.78)				81 (0.79)	
HSV-tk coding			16 (.13)				6 (.07)				10 (.08)				3 (0.04)	
Rearranged			43 (.35)				52 (.60)				17 (.14)				19 (0.18)	

[a]Numbers of mutants observed. Numbers in parentheses indicate 1-unit, 2-unit events

[b]Proportion of total events

[c]Observed a 4-unit deletion

[d]Observed a 4-unit insertion

[e]Observed a 5-unit deletion

n.d. - not determined

Table S3 Microsatellite mutation rate and sequence data for independent clones in PMS2-Deficient LCL1261 Cells.

	GT/CA_{10}			GT/CA_{13}			GT/CA_{16}			GT/CA_{19}		
	Mutation Rate	Insertion[a]	Deletion[a]	Mutation Rate	Insertion	Deletion	Mutation Rate	Insertion	Deletion	Mutation Rate	Insertion	Deletion
Clone A	4.30E-05	13 (13,0)	2 (2,0)	3.50E-04	21 (21,0)	0	1.38E-03	19 (19,0)	0	9.16E-03	17 (17,0)	0
Clone B	5.60E-05	14 (14,0)	2 (1,1)	4.67E-04	11 (11,0)	0	6.20E-04	22 (22,0)	0	5.29E-03	17 (7,0)[c]	0
Clone C	1.00E-04	16 (16,0)	0	7.48E-04	21 (21,0)	0	4.24E-03	21 (21,0)	0	2.66E-03	15 (15,0)	0
Clone D										1.08E-02	8 (8,0)	0
Totals, by type[b]												
Microsatellite		47 (.70)			53 (.78)			62 (.95)			57 (.98)	
HSV-tk coding		14 (.21)			15 (.22)			3 (.05)			1 (.02)	
Rearranged		6 (.09)			0			0			0	

[a]Numbers of mutants observed. Numbers in parentheses indicate 1-unit, 2-unit events

[b] Summation for all clones. Proportion of total events shown in parentheses

[c]Observed a 4-unit insertion
