# Supplementary material for: Mature Microsatellites: Mechanisms Underlying Dinucleotide Microsatellite Mutational Biases in Human Cells
Source: G3 (Bethesda). 2013 Mar 1;3(3):451–63. doi: 10.1534/g3.112.005173 (PMC3583453; doi:10.1534/g3.112.005173)
Supplement: Supporting Information [file supp_3.3.451_FigureS1.pdf]

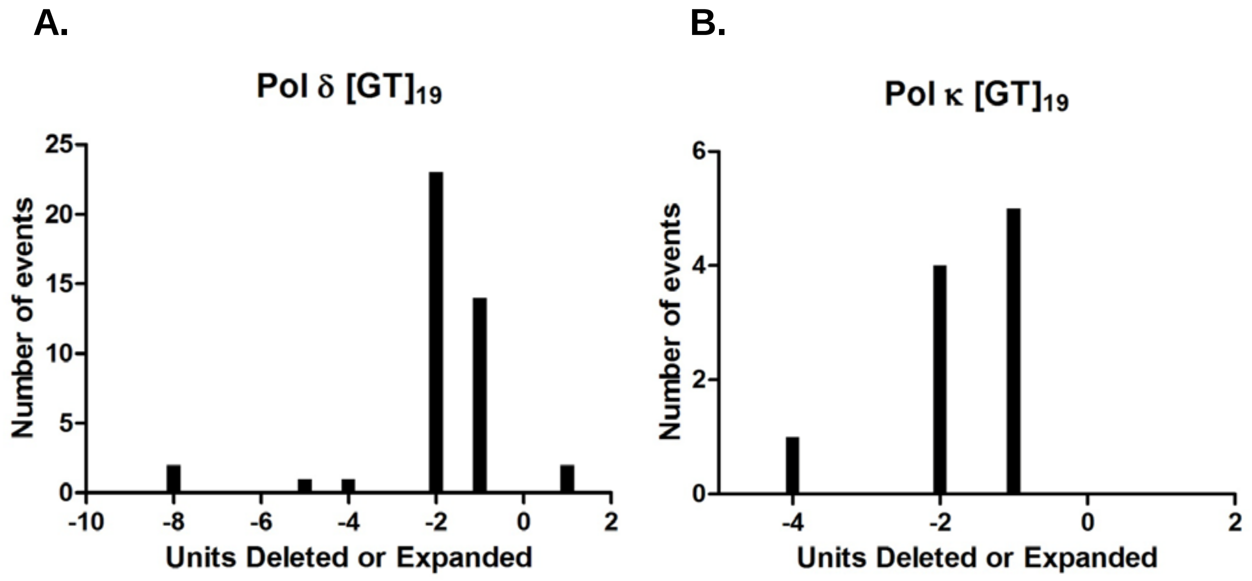

Figure S1 Pol δ (A) and pol κ (B) replicate $[GT]_{19}$ microsatellite allele with substantial deletion bias
