# Supplementary material for: Mature Microsatellites: Mechanisms Underlying Dinucleotide Microsatellite Mutational Biases in Human Cells
Source: G3 (Bethesda). 2013 Mar 1;3(3):451–63. doi: 10.1534/g3.112.005173 (PMC3583453; doi:10.1534/g3.112.005173)
Supplement: Supporting Information [file supp_3.3.451_TableS3.pdf]

Table S3 Microsatellite mutation rate and sequence data for independent clones in PMS2-Deficient LCL1261 Cells.

	GT/CA$_{10}$			GT/CA$_{13}$			GT/CA$_{16}$			GT/CA$_{19}$		
	Mutation Rate	Insertion[a]	Deletion[a]	Mutation Rate	Insertion	Deletion	Mutation Rate	Insertion	Deletion	Mutation Rate	Insertion	Deletion
Clone A	4.30E-05	13 (13,0)	2 (2,0)	3.50E-04	21 (21,0)	0	1.38E-03	19 (19,0)	0	9.16E-03	17 (17,0)	0
Clone B	5.60E-05	14 (14,0)	2 (1,1)	4.67E-04	11 (11,0)	0	6.20E-04	22 (22,0)	0	5.29E-03	17 (7,0)[c]	0
Clone C	1.00E-04	16 (16,0)	0	7.48E-04	21 (21,0)	0	4.24E-03	21 (21,0)	0	2.66E-03	15 (15,0)	0
Clone D										1.08E-02	8 (8,0)	0
Totals, by type[b]												
Microsatellite		47 (.70)			53 (.78)			62 (.95)			57 (.98)	
HSV-tk coding		14 (.21)			15 (.22)			3 (.05)			1 (.02)	
Rearranged		6 (.09)			0			0			0	

[a]Numbers of mutants observed. Numbers in parentheses indicate 1-unit, 2-unit events

[b] Summation for all clones. Proportion of total events shown in parentheses

[c]Observed a 4-unit insertion
